# Hemolymph protein profiles of subterranean termite *Reticulitermes flavipes* challenged with methicillin resistant *Staphylococcus aureus* or *Pseudomonas aeruginosa*

**DOI:** 10.1038/s41598-018-31681-2

**Published:** 2018-09-05

**Authors:** Yuan Zeng, Xing Ping Hu, Guanqun Cao, Sang-Jin Suh

**Affiliations:** 1 Department of Entomology and Plant Pathology, Auburn University, Auburn, AL USA; 2 Department of Mathematics and Statistics, Auburn University, Auburn University, Auburn, AL USA; 3 Department of Biological Sciences, Auburn University, Auburn University, Auburn, AL USA; 4 Present Address: Department of Bioagricultural Sciences and Pest Management, Colorado State University, Fort Collins, CO USA

## Abstract

When the subterranean termite *Reticulitermes flavipes* is fed heat-killed methicillin resistant *Staphylococcus aureus* (MRSA) or *Pseudomonas aeruginosa*, the termite produces proteins with antibacterial activity against the inducer pathogen in its hemolymph. We used a proteomic approach to characterize the alterations in protein profiles caused by the inducer bacterium in the hemolymph of the termite. Nano-liquid chromatography-tandem mass spectrometry analysis identified a total of 221 proteins and approximately 70% of these proteins could be associated with biological processes and molecular functions. Challenges with these human pathogens induced a total of 57 proteins (35 in MRSA-challenged, 16 in *P*. *aeruginosa*-challenged, and 6 shared by both treatments) and suppressed 13 proteins by both pathogens. Quasi-Poisson likelihood modeling with false discovery rate adjustment identified a total of 18 and 40 proteins that were differentially expressed at least 2.5-fold in response to MRSA and *P*. *aeruginosa*-challenge, respectively. We selected 7 differentially expressed proteins and verified their gene expression levels via quantitative real-time RT-PCR. Our findings provide an initial insight into a putative termite immune response against MRSA and *P*. *aeruginosa*-challenge.

## Introduction

Insect hemolymph plays key roles in insect innate immunity^1^. Although many of the hemolymph components have yet to be characterized, some of the proteins have been identified and their functions elucidated. For example, hexamerins and arylphorins have been determined to be the source of amino acids for components of insect cuticles; lipophorins and other related enzymes (e.g. esterases, lipases) function in lipid transportation and hydrolysis; vitellogenins play an important role in embryo development; and cytokines are involved in intercellular communications. In addition, the hemolymph contains components of the insect immune system, including effector molecules such as cytokines, enzyme cascades, and antioxidant proteins, demonstrating that it is vital for defense against pathogens or tissue damage^2–4^.

Proteomics is an important tool for studying changes in insect hemolymph proteome during development and in response to environmental effects^5–7^. Previous studies have demonstrated changes in the hemolymph proteomes of fruit fly, silkworm, white butterfly, and tobacco hornworm in response to immune challenges^6,8–10^. In termites, Liu *et al*.^11^ identified differential expression of 20 hemolymph proteins and upregulated expression of 3 immune genes in the subterranean termite *Reticulitermes chinensis* when challenged with an entomopathogenic fungus *Metarhizium anisopliae*. However, very little is known about the effect of bacterial challenge on the termite hemolymph proteome.

The eastern subterranean termite *Reticulitermes flavipes* (Kollar) is widely distributed in the United States and causes serious damage to structures and plants to result in significant economic losses^12^. Subterranean termites have evolved various defense strategies against pathogens as they nest and forage in microbe-rich soil^13^. We have previously reported the existence of a broad-spectrum of constitutive activity against a variety of Gram-positive and Gram-negative bacteria, including human pathogens, in the hemolymph of *R*. *flavipes*^14,15^. Interestingly, although the naïve termite hemolymph was active against the human pathogen *S*. *aureus*, it was inactive against MRSA, the methicillin resistant derivative of *S. aureus*, that has emerged as one of the most dangerous Gram-positive pathogens. The hemolymph from naïve termite was also inactive against *Pseudomonas aeruginosa*, one of the most dangerous Gram-negative nosocomial pathogens. However, the termite developed inducible anti-MRSA or anti-*P*. *aeruginosa* activity in its hemolymph when it was fed heat-killed MRSA or *P*. *aeruginosa*, respectively^14^. The induction of anti-MRSA or anti-*P. aeruginosa* activity affected the overall antimicrobial spectrum of the *R. flavipes* hemolymph. MRSA-challenged termite hemolymph gained anti-MRSA activity while maintaining activity against two other Gram-positive bacteria, including *Streptococcus pyogenes*, but completely lost activity against three Gram-negative bacteria tested. *P. aeruginosa*-challenged termites gained anti-*P. aeruginosa* and anti-MRSA activity while maintaining activity against other bacteria tested. As an initial step towards elucidating the inducible antimicrobial defense mechanisms of *R*. *flavipes*, we determined the hemolymph protein profiles of termites challenged with either MRSA or *P*. *aeruginosa*.

## Results

### Analysis of hemolymph proteome

**Figure 1:**
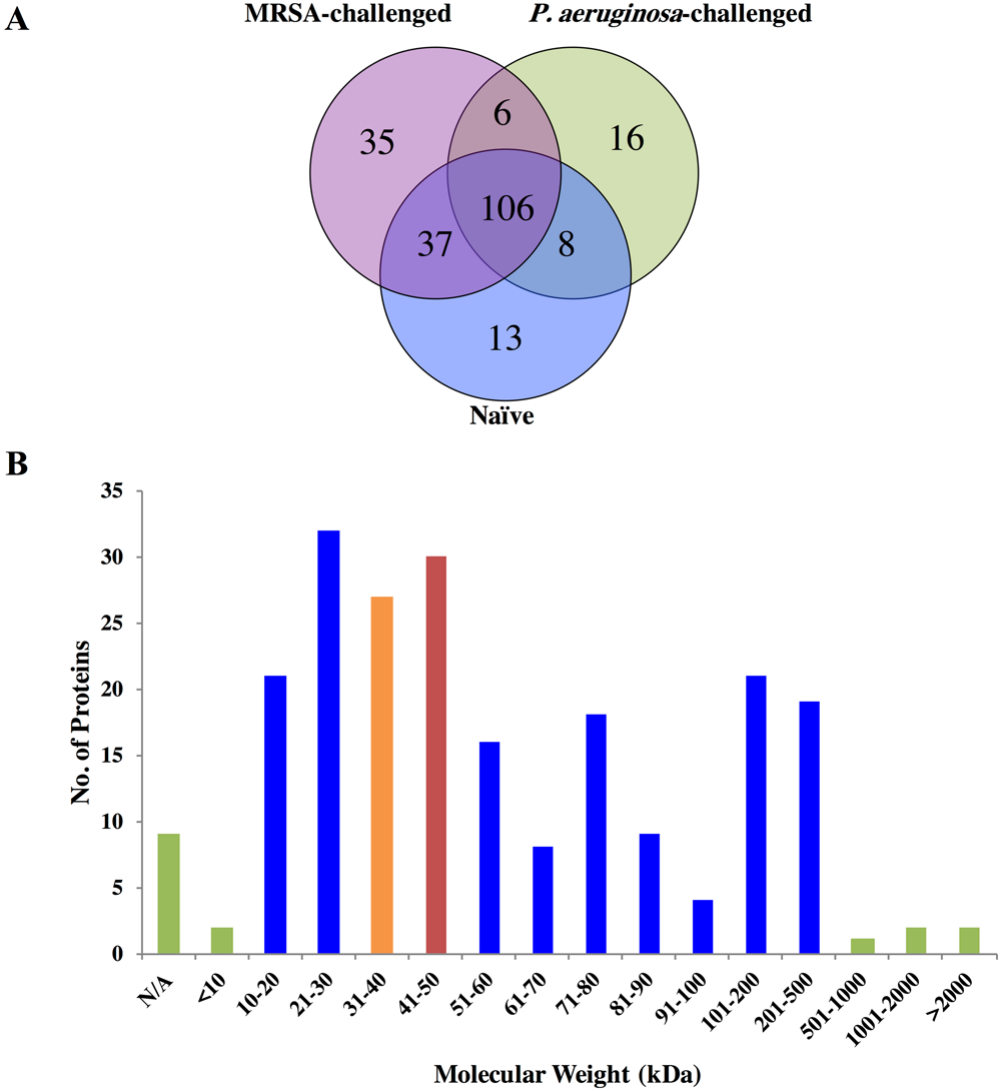
Summary of the identified hemolymph proteins in bacterial pathogen-challenged and naïve *R. flavipes* workers. (**A**) Comparison of proteins identified in naïve and bacterial pathogen-challenged *R. flavipes*. (**B**) Size distribution of proteins.

NCBI protein database search showed that 22,338 spectra matched those of trypsin digested termite hemolymph proteins at ≥98% probabilities to achieve 0.10% Decoy False Discovery Rate (FDR). The matching spectra corresponded to 181 clusters containing a total of 221 proteins with at least two peptide matches at ≥95% confidence. Of the 221 proteins, 106 proteins were present in all three termite groups, 37 proteins were present in the naïve and the MRSA-challenged termites, 8 proteins were present in the naïve and the *P. aeruginosa*-challenged termites, and 6 proteins were present only in the two bacteria-challenged insects. In summary, two bacteria induced the production of 57 proteins but reduced the production of 13 proteins. Of the 57 induced proteins, 35 were detected only in the MRSA-challenged, 16 in the *P. aeruginosa*-challenged, and 6 in both bacteria-challenged termite groups, respectively (Fig. 1A). Approximately 68% of the proteins had molecular weight (MW) between 10 to 80 kDa (Fig. 1B). The smallest protein was 8 kDa (antioxidant enzyme; AGM32333.1) and the largest protein was 2,068 kDa (uncharacterized protein; XP_014096235.1).

**Figure 2:**
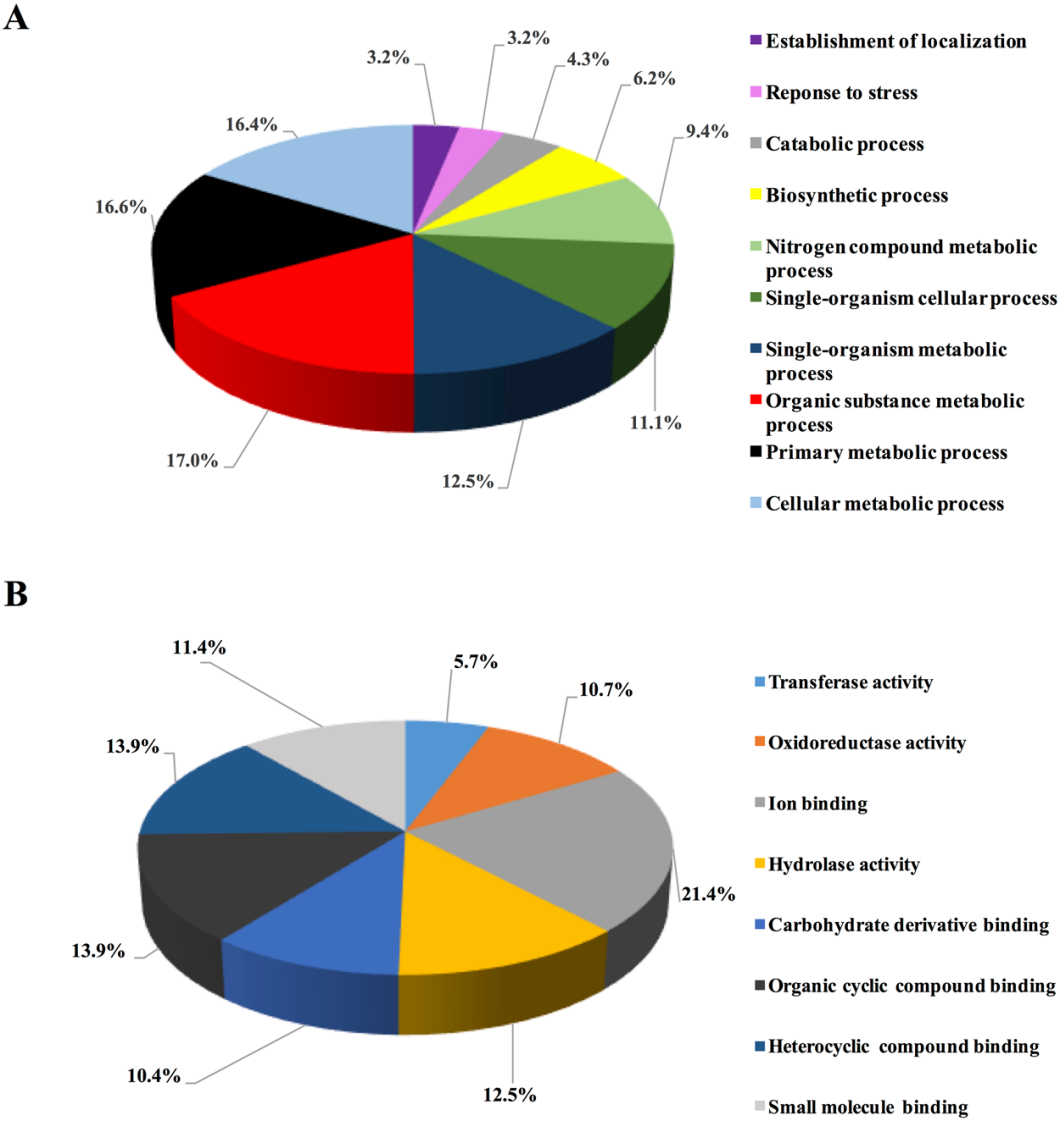
Protein categorization by gene ontology (GO). (**A**) GO based on biological processes. (**B**) GO based on molecular functions. The percentages indicate relative abundances.

Blast2Go analysis associated many of the hemolymph protein sequences (70%) with a variety of biological processes (GO:0008150) and molecular functions (GO:0003674). The identified proteins were annotated into 10 categories based on biological processes (Fig. 2A) or 8 categories based on molecular functions (Fig. 2B). A comparison among relative abundances of sub-levels of annotated categories on biological process showed that hemolymph proteins that participate in organonitrogen compound metabolism (GO:1901564), macromolecule metabolism (GO:0043170), carbohydrate metabolism (GO:0005975), phosphorus metabolism (GO:0006793), redox reactions (GO:0055114), small molecule metabolism (GO:0044281), and transport (GO:0006810) had a higher abundance. A comparison among relative abundances of sub-levels of annotated molecular functions showed termite hemolymph proteins involved in nucleotide binding (GO:0000166), purine nucleoside binding (GO:0001883), ribonucleoside binding (GO:0032549), and metal ion binding (GO:0046872) had a higher abundance than those annotated with catalytic activities.

**Figure 3:**
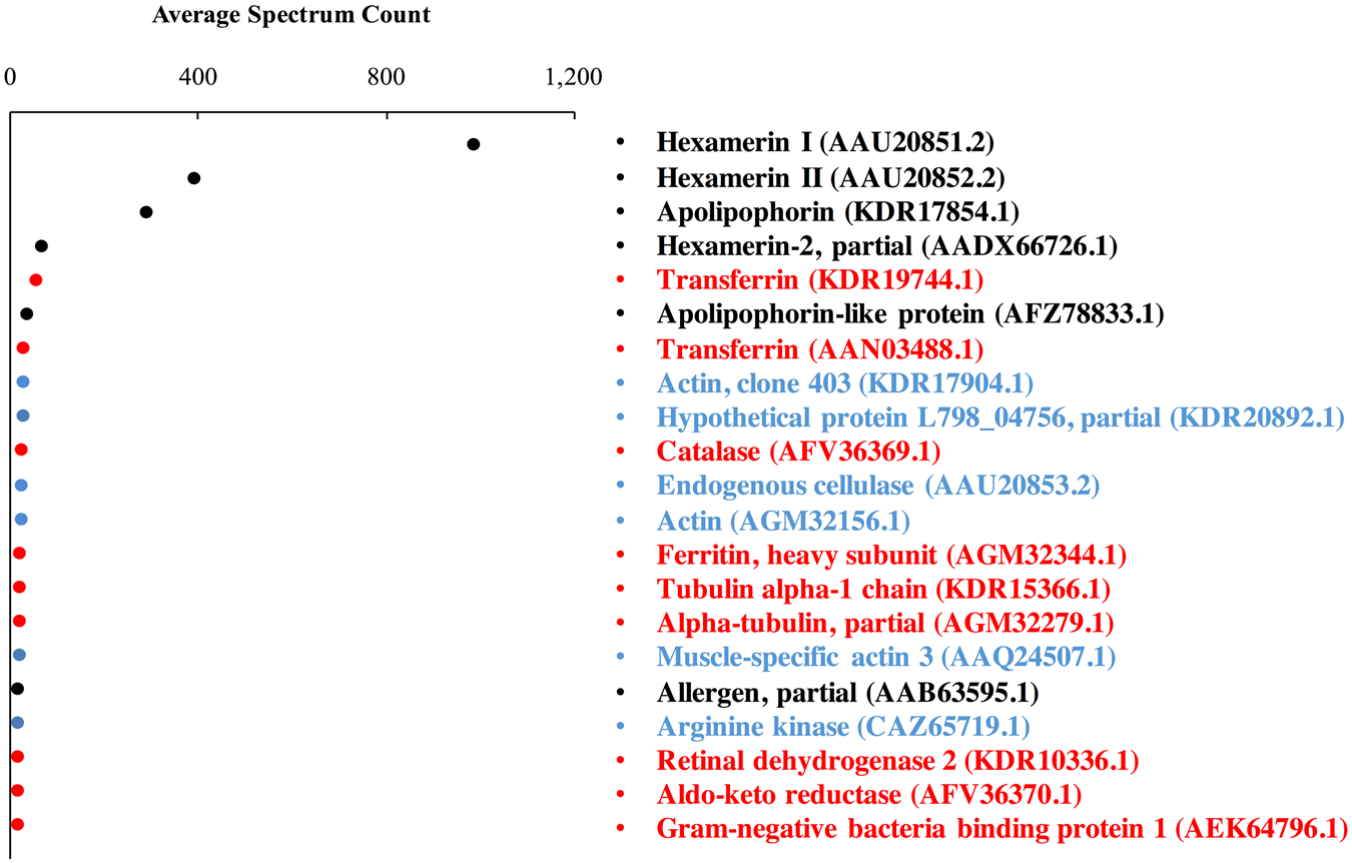
Twenty-one most abundant proteins in three *R. flavipes* hemolymph samples. The abundance value of each protein was estimated as average spectrum count. Colors show the protein category: storage protein, black; immune-related protein, red; other proteins, blue.

According to the total spectral count, the 21 most highly expressed hemolymph proteins were divided into three categories of 6 storage proteins (hexamerin I, hexamerin II, hexamerin-2, apolipophorins, apolipophorin-like protein, and allergen), 9 immune-related proteins (2 transferrins, catalase, ferritin, 2 alpha-tubulin, retinal dehydrogenase 2, aldo-keto reductase, and gram-negative bacteria binding protein), and 6 other proteins (3 actins, hypothetical protein L798_04756, endogenous cellulase, and arginine kinase) (Fig. 3).

### Effect of bacterial pathogen-challenge on hemolymph proteome

#### Alterations in the hemolymph proteome following MRSA-challenge

MRSA challenge induced production of 41 proteins and reduced production of 21 proteins (Fig. 1A). Of these, 4 proteins were induced at least 2.5-fold and 14 proteins were reduced at least 2.5-fold (Table 1). Of the induced (upregulated) proteins, transferrin, has been associated with antimicrobial activity as a part of the immune response. Among the reduced (downregulated) proteins, 6 are associated with immune response (i.e., response to stress, cytoskeletal modeling, detoxification, and immune effectors). These molecules include beta-glucosidase, papilin, c-type lysozyme, apolipophorin, peroxiredoxin-6, and cathepsin L-like protein. Thus, exposure to heat-killed MRSA up-regulated one but down-regulated 6 known immune proteins in *R. flavipes*.

**Table 1:** Differentially expressed hemolymph proteins of R. flavipes following MRSA-challenge.

Protein	Accession Number	Poisson.FDR *p*-value	Quasi.FDR *p*-value	Rate Ratio*
transferrin	AAQ62963.2	0.0909	0.0193	32.91
calponin-likey domain containing protein	AGM32561.1	0.0909	0.0193	32.91
predicted: myosin heavy chain, muscle isoform X26	XP_014282764.1	0.1375	0.0308	32.59
predicted: ATP synthase subunit alpha	XP_015124302.1	0.1375	0.0308	32.59
protein yellow	KDR22429.1	0.0696	<0.0001	−34.62
multifunctional protein ADE2, partial	KDR09851.1	0.0018	0.0008	−34.29
predicted: alpha, alpha-trehalose-phosphate synthase [UDP-forming]-like isoform X1	XP_015365044.1	0.1938	<0.0001	−33.62
cytosolic carboxypeptidase-like protein 5, partial	KDR22169.1	0.1938	<0.0001	−33.62
multifunctional protein ADE2	EZA56375.1	0.1938	<0.0001	−33.62
predicted: bifunctional purine biosynthesis protein PURH	XP_015365809.1	0.0909	0.0193	−32.91
hypothetical protein L798_01615	KDR07960.1	0.0909	0.0193	−32.91
cathepsin L-like protein	AGM32335.1	0.0909	0.0193	−32.91
lambda-crystallin-like protein	KDR11934.1	0.1375	0.0308	−32.59
peroxiredoxin-6	KDR10377.1	0.1375	0.0308	−32.59
apolipophorin	KDR18107.1	0.0272	0.0308	−32.15
c-type lysozyme-2	AFZ78837.1	0.0696	0.0469	−31.73
beta-glucosidase	BAO85044.1	0.1375	0.0308	−2.00
papilin	KDR22055.1	0.0049	0.0308	−1.79

*Rate ratio: −1.32 ≤ log_2_(rate1/rate2) ≤ 1.32.

**Figure 4:**
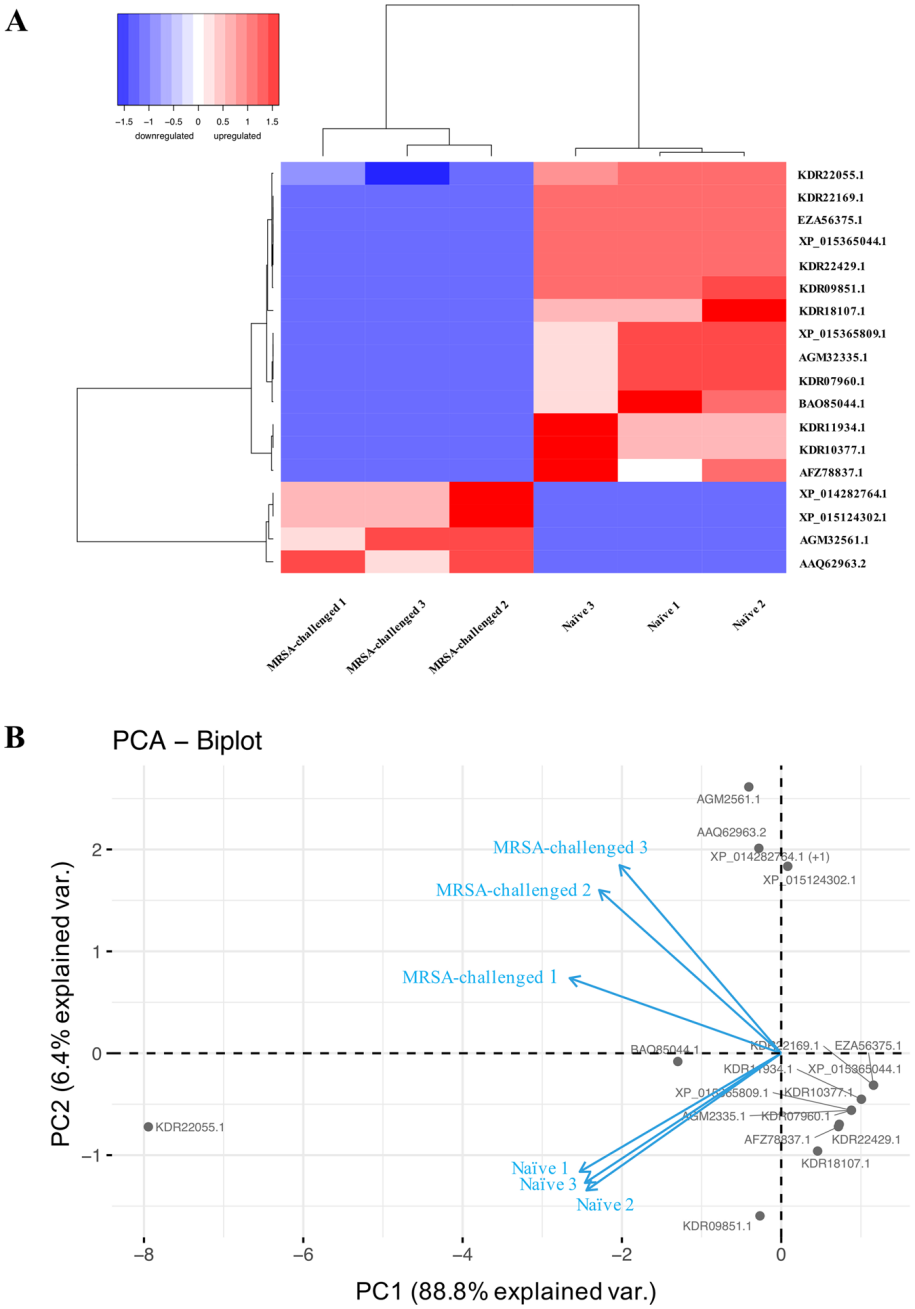
Hierarchical clustering analysis (**A**) and principal component analysis (**B**) based on 18 proteins significantly changed in abundances between MRSA-challenged and naïve termites within the dataset. (**A**) Both samples and proteins were clustered using Ward’s method, and with Pearson correlation as similarity metric. The samples are shown horizontally (columns) and the proteins are shown vertically (rows). The dendrograms represent the distances between clusters. Protein expression levels are represented in the color scale of blue (downregulated) to red (upregulated). (**B**) Grey dots and blue arrows represent hemolymph proteins and variables, respectively. Each axis represents a principal component (PC1 and PC2) with the percentage of the total variance it explains.

Hierarchical clustering based on the expression profile of the 18 discriminatory proteins (4 upregulated and 14 downregulated) separated samples into two main clusters corresponding to the MRSA-challenge (Fig. 4A). Similarity in protein expression profiles clustering the 6 samples was summarized in a biplot by the principle component analysis (PCA) (Fig. 4B). Both principal components (PC1 and PC2) explained a total variation of 95.2%. The first principal component (PC1) differentiated MRSA-challenged and naïve termite hemolymph samples, while the second principal component (PC2) was a measure of antibacterial peptide and detoxification enzyme (e.g. c-type lysozyme 2, peroxiredoxin-6) across all 18 discriminatory proteins.

#### Alterations in the hemolymph proteome following *P. aeruginosa*-challenge

The *P. aeruginosa*-challenge resulted in differential accumulation of 40 proteins with at least 2.5-fold changes (Table 2). These proteins were annotated to metabolism, development, stress response, immune signaling, immune effectors and other functions. Of the 4 upregulated proteins, 2 immune response related proteins (actin^16,17^ and alpha-tubulin, partial^18,19^) levels were increased at least 2.5-fold. In contrast, *P. aeruginosa*-challenge downregulated 36 proteins and 24 of them completely disappeared from the proteome. Most of the downregulated proteins are involved in metabolic processes and stress response. Of the downregulated proteins, 9 have been reported as immune-related proteins including ferritin, alcohol dehydrogenase, glutathione S-transferase, aldo-keto reductase, regucalcin, c-type lysozyme, Cu/Zn superoxide dismutase, hemocytin, and prostaglandin reductase^20–25^.

**Table 2:** Differentially expressed hemolymph proteins of *R. flavipes* following *P. aeruginosa*-challenge.

Protein	Accession Number	Poisson.FDR *p*-value	Quasi.FDR *p*-value	Rate Ratio*
enolase	AGM32398.1	0.0034	<0.0001	35.20
malate dehydrogenase	KDR14372.1	0.0061	0.0020	33.59
alpha tubulin, partial	AGM32279.1	<0.0001	0.0045	2.50
actin	AGM32156.1	0.0053	0.0218	1.61
ferritin	AGM32322.1	<0.0001	0.0007	−35.55
glutamine synthetase 2 cytoplasmic	KDR18484.1	0.0034	<0.0001	−35.20
uncharacterized protein	AGM32706.1	0.0198	<0.0001	−34.62
translationally-controlled tumor protein-like protein	EZA58324.1	0.0198	<0.0001	−34.62
protein yellow	KDR22429.1	0.0198	<0.0001	−34.62
alcohol dehydrogenase	XP_014291627.1	0.0198	<0.0001	−34.62
selenium-binding protein 1-A	KDR09028.1	0.0001	0.0017	−34.50
multifunctional protein ADE2, partial	KDR09851.1	0.0003	0.0005	−34.29
multifunctional protein ADE2	EZA56375.1	0.1132	<0.0001	−33.62
cytosolic carboxypeptidase-like protein 5, partial	KDR22169.1	0.1132	<0.0001	−33.62
predicted: alpha,alpha-trehalose-phosphate synthase [UDP-forming]-like isoform X1	XP_015365044.1	0.1132	<0.0001	−33.62
aldo-keto reductase 1	AMJ21949.2	0.0061	0.0020	−33.59
beta-glucuronidase	KDR08779.1	0.0061	0.0020	−33.59
teneurin-3	KDR07188.1	0.0002	0.0030	−32.96
unknown	AEE63607.1	0.0351	0.0077	−32.91
hypothetical protein L798_01615	KDR07960.1	0.0351	0.0077	−32.91
regucalcin	KDR12743.1	0.0351	0.0077	−32.91
predicted: bifunctional purine biosynthesis protein PURH	XP_015365809.1	0.0351	0.0077	−32.91
SCP-like extracellular domain containing protein 2	AGM32430.1	0.0011	0.0069	−32.61
c-type lysozyme-2	AFZ78837.1	0.0198	0.0223	−31.73
filamin-B	EZA55995.1	0.0198	0.0223	−31.73
Ribose-phosphate pyrophosphokinase 2, partial	KDR10178.1	0.0198	0.0223	−31.73
neurotrypsin	KDR22858.1	0.0198	0.0223	−31.73
predicted: filamin-A isoform X1	XP_015127256.1	0.0198	0.0223	−31.73
Cu/Zn superoxide dismutase	AGM32998.1	0.0121	0.0405	−2.66
enolase	AGM32397.1	0.0147	0.0456	−2.58
hemocytin, partial	KDR23192.1	0.0361	0.0384	−2.46
synaptic vesicle membrane protein VAT-1-like protein-like, partial	KDR16462.1	0.0128	0.0411	−2.42
hypothetical protein L798_04756, partial	KDR20892.1	0.0000	0.0004	−2.38
prostaglandin reductase 1	KDR24385.1	0.0627	0.0150	−2.00
hypothetical protein L798_11509	KDR14754.1	0.0034	0.0217	−1.95
glutathione S-transferase	AFZ78680.1	0.0927	0.0045	−1.87
papilin	KDR22055.1	0.0019	0.0047	−1.66
pasma alpha-L-fucosidase	KDR21959.1	0.0198	0.0079	−1.58
beta-ureidopropionase	KDR12152.1	0.0198	0.0450	−1.53
putative chemosensory protein	BAU20278.1	0.1696	0.0473	−1.46

*Rate ratio: −1.32 ≤ log_2_(rate1/rate2) ≤ 1.

**Figure 5:**
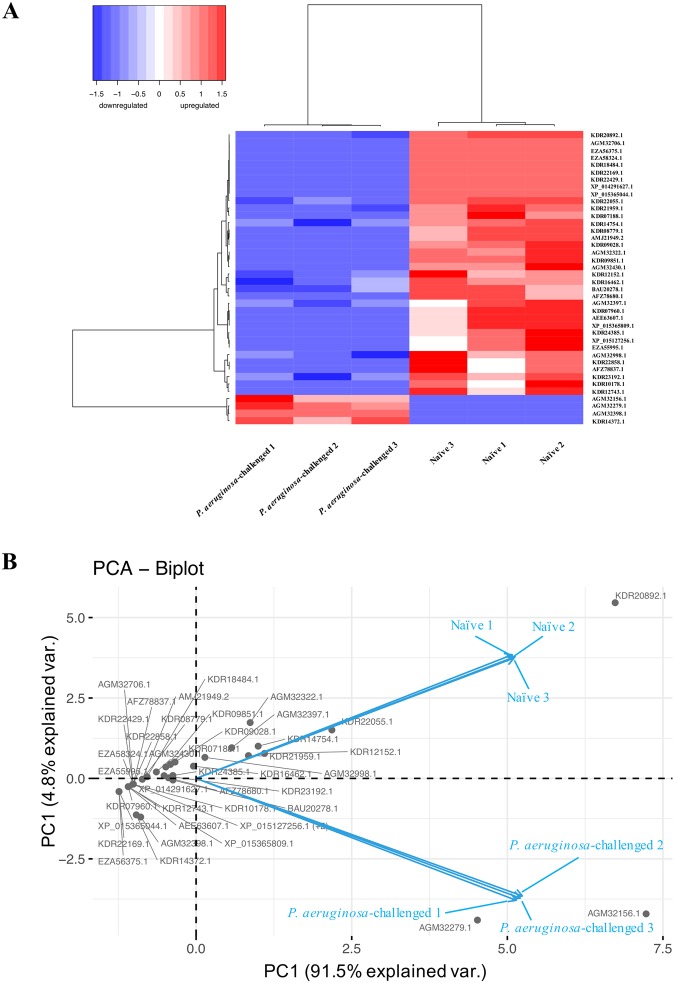
Hierarchical clustering analysis (**A**) and principle component analysis (**B**) based on 40 proteins significantly changed in abundances between *P. aeruginosa*-challenged and naïve termites within the dataset. (**A**) Both samples and proteins were clustered using Ward’s method, and with Pearson correlation as similarity metric. The samples are shown horizontally (columns), the proteins vertically (rows). The dendrograms represent the distances between clusters. Protein expression levels are represented in the color scale of blue (downregulated) to red (upregulated). (**B**) Grey dots and blue arrows represent hemolymph proteins and variables, respectively. Each axis represents a principal component (PC1 and PC2) with the percentage of the total variance it explains.

Hierarchical clustering (Fig. 5A) of these 40 discriminatory proteins in *P. aeruginosa*-challenged and naïve termites essentially separated samples into two main clusters. PCA analysis by the first two principal components explained 96.3% of the variations (Fig. 5B). The first principal component (PC1) differentiated *P. aeruginosa*-challenged and naïve termite hemolymph samples, and the second principal component (PC2) is a measure of enzymes involved in metabolism (glutamine synthetase 2)^26^, detoxification (glutathione S-transferase)^20^, cell migration (filamin A)^27^, and antibacterial defenses (c-type lysozyme 2)^28^.

### RT-qPCR verification of selected protein levels

**Figure 6:**
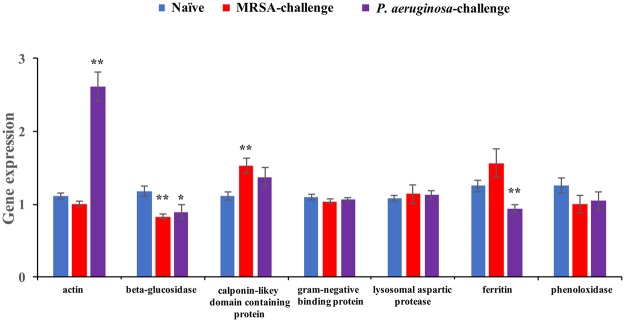
Transcript levels of genes encoding for seven selected proteins (mean ± SE) between bacteria-challenged and naïve termites. Asterisks denote significant differences between bacteria-challenged and naïve termites. (*p < 0.05; **p < 0.01; paired t-test, n = 9, α = 0.05).

Alterations in protein levels can be due to various factors including gene expression and changes in protein turn-over. We verified our proteome data by determining the transcript levels of seven selected genes via RT-qPCR (Supplementary Table 1) with the gene encoding alpha-tubulin 2 as the internal control. These genes were selected because they were expected to be involved in the insect’s immune responses or hormone regulation. As determined from the proteome analysis, MRSA-challenge significantly downregulated the expression level of the beta-glucosidase gene (*t* = 4.2846, df = 8, *p* = 0.0011) and induced calponin-likey domain containing protein gene (*t* = −3.4864, df = 8, *p* = 0.0043) expression when compared with those in the naïve termites (Fig. 6). *P. aeruginosa*-challenge increased the expression level of actin gene (*t* = −7.3798, df = 8, *p* < 0.0001) while reduced the expression levels of ferritin encoding gene (*t* = 3.2076, df = 8, *p* = 0.0067) and beta-glucosidase gene (*t* = 2.3772, df = 8, *p* = 0.0313) (Fig. 6). Expression of the other genes remained similar in the naïve versus MDR-challenged termites to support the proteome data (Fig. 6; Tables 1 and 2).

## Discussion

In order to further understand the inducible anti-MRSA and anti-*P. aeruginosa* activity in *R. flavipes*, we analyzed the hemolymph proteomes of the naïve and the bacterial pathogen-challenged *R. flavipes* workers in this study. We used nano-LC-MS/MS to analyze the hemolymph proteome and identified a total of 221 proteins. Of these, 57 proteins were induced by bacterial pathogen-challenges and 164 were constitutively present in naïve termites. The abundance of hexamerins, apolipophorin, and actins in termite hemolymph suggests that hemolymph is a source for nutrient and ion storage and transportation.

Three immune effector proteins, phenoloxidase (PO), Gram-negative binding protein (GNBP) and lysosomal aspartic protease were constitutively present in all three samples and their expression levels remained constant after bacterial pathogen-challenges (data not shown). POs play a crucial role in formation of melanin and reactive oxygen species (ROS) to defend against microbes and other parasites in arthropods^29–31^. GNBPs possess antimicrobial activity with the β-1,3-glucanase activity^32^. Lysosomal aspartic protease, which has been documented in human and various insects^33–35^, may contribute to the overall bactericidal activity^36^. Therefore, it is likely that these molecules are part of the constitutive antibacterial activities we previously reported for *R. flavipes*^14,15^. However, unlike in other studies in which transcript levels of the genes that encode for POs and GNBPs were overexpressed after immune challenge^11,37,38^, these transcripts and the proteins remained unchanged following MRSA or *P. aeruginosa*-challenge in our study. This suggests that the anti-MRSA and anti-*P. aeruginosa* activities in the hemolymphs of challenged *R. flavipes* are not due to these previously characterized enzymes.

Some of the differentially expressed proteins in response to bacteria-challenges are involved in immune-related processes including cytoskeletal modeling, iron metabolism, antioxidant-related immune response, stress response, and immune effectors. In addition, enzymes such as enolase and malate dehydrogenase of central metabolic pathways^39,40^ were upregulated in *P. aeruginosa*-challenged termites to suggest a higher energetic demand of the host to suppress *P. aeruginosa* infection. This is supported by recent studies in mosquitos (*Anopheles gambiae*)^41^ after a parasite infection and moths (*Galleria mellonella*)^42^ to bacterial and fungal infections. Interestingly, in termites challenged with heat-killed MRSA, neither the enolase nor the malate dehydrogenase was overexpressed compared to the naïve termites. In contrast, a lipid transporter and an energy storage protein (i.e., apolipophorin) were decreased suggesting downregulation of the lipid metabolism in response to MRSA-challenge^43^.

Successfully competing for iron is vital for preventing pathogen survival in the hosts^44,45^. In support of this strategy, transferrin was upregulated in MRSA-challenged termites^46–48^. In contrast to transferrin, a c-type lysozyme with hydrolase activity was severely downregulated by MRSA-challenge. Given the activity of lysozyme in hydrolyzing the β-1,4 linkage of peptidoglycan in bacterial cell wall and its importance as an antibacterial enzyme, it is curious that a c-type lysozyme disappeared in MRSA-challenged termites.

In *P. aeruginosa*-challenged termites, two proteins that had previously been associated with immune response were induced. Actin and alpha-tubulin are cytoskeletal elements that help maintain cell shape, participate in cellular division, and intracellular transport of molecules^17,20,49–51^. Expression of genes encoding for actin and alpha-tubulin have been reported to be upregulated in various insects in response to bacterial challenge^7,52^. In comparison to the MRSA-challenged termites, the abundance of actin and tubulin was significantly higher in *P. aeruginosa*-challenged termites (data not shown). A recent study demonstrated that insect actin identified in mosquito can mediate bacterial cell killing through phagocytosis or direct antibacterial action when it binds to the surface of bacterial cells^16^. Thus, it is possible that actin is an antibacterial molecule that inhibits *P. aeruginosa* and other susceptible bacteria^14^ in *R. flavipes*’ hemolymph.

We hypothesized more proteins associated with insect immune response should be upregulated after a 24 h bacterial pathogen-challenge. However, more proteins, including those that have been previously reported to play roles in stress response and immunity, were downregulated in our study. Our results are similar to the study in *D. melanogaster* larvae hemolymph^40^ in which enzymes/proteins involved in metabolism (carbohydrate, energy, lipid, and protein metabolisms) and the overall stress response (heat shock, immune response, and detoxification) were down-regulated 24 h following a bacterial-challenge. In addition, a previous study in *R. chinensis* reported an equal number of stress response proteins were either upregulated or downregulated following an entomopathogenic fungus induction^11^.

We selected 7 proteins to verify the LC-MS/MS data by RT-qPCR. As described in the results, our RT-qPCR data validated the upregulated level of actin and downregulated levels of ferritin and beta-glucosidase in *P. aeruginosa*-challenged termites. We also verified that the upregulated level of calponin-likey domain containing protein and the reduction of beta-glucosidase in MRSA-challenged termites were due to differential gene expression at the transcriptional level. Although ferritin, an iron storage and transport protein, is important for oxidative stress, a significantly lower level of this protein was observed in termites after *P. aeruginosa* challenge in contrast to several previous studies^20,53,54^. However, in support of our data, a recent study reported a negative correlation between an increase of ferritin with the survivability of a lepidopteran insect against bacterial infection^42^. The expression of a digestive enzyme, beta-glucosidase, was also decreased after the 24 h bacterial pathogen-challenge. This suggests that termites may reduce secretion of proteins involved in cellulose degradation to couple with immune defense although cellulase abundance remained similar among treatments to support the metabolic demand (data not shown). Our data support a model in which the host responds to bacteria not only by producing proteins that can fight the potential infection directly but also by engaging its metabolic pathways and energy resources required to support the effort^18^.

The present study demonstrated that heat-killed MRSA or *P. aeruginosa* alters the proteome profile in the eastern subterranean termite hemolymph 24 h post bacterial feeding. The differential expression of proteins involved in metabolic activity and in insect immunity among naïve, MRSA-challenged and *P. aeruginosa* suggest that termite host defense against bacterial challenge is a concerted response involving proteins that directly kill the microbes as well as proteins in stress response and metabolism that indirectly support the host’s defensive effort. Moreover, the identified immune effectors provide insights for the potential new targets of antimicrobial discovery against *P. aeruginosa* and MRSA. In conclusion, by using a proteome-wide scale study on the eastern subterranean termites where immunity and protein energy flux are tightly coupled, we provide an initial insight into a putative termite immune response against two human bacterial pathogens.

## Methods

### Termite collection and hemolymph extraction

*R. flavipes* workers were collected on the Auburn University campus as previously described^14^ and workers were reared with filter papers (Whatman #1) in Urban Entomology Laboratory at 25 ± 2 °C for at least 20 days. Three groups of 4-g termite workers were introduced into Petri plates (15 cm × 2.5 cm), surface sterilized with 70% ethanol immediately before being subjected to experiments. One group was fed with a sterile filter paper (Whatman #1) moistened with 3 ml of milli-Q (MQ) water as control. The other two groups were challenged by feeding the termites with the same filter paper moistened with 3 ml of heat-killed *P. aeruginosa* or MRSA suspension, respectively. Each bacterial suspension contained approximately 1.8 × 10^9^ cells. Termites were harvested after 24 h feeding and the cell-free hemolymph was extracted using the method described in Zeng *et al*.^14^. For each treatment, termite immunization and hemolymph extraction were performed in triplicates.

### Proteomic analysis

Nano LC-MS/MS analyses were performed by the Mass Spectrometry & Proteomics Resource of the W. M. Keck Foundation Biotechnology Resource Laboratory of Yale School of Medicine. Cell-free hemolymph extracts were processed by reduction, alkylation and trypsin digestion for nano LC-MS/MS analysis. Briefly, the hemolymph extracts were dried and reconstituted in 40 μl 8 M urea, 0.4 M NH_4_HCO_3;_ reduced in 4.0 μl 45 mM dithiothreitol (DTT), and incubated 30 min at 37 °C before alkylation in 4.0 μl 100 mM iodoacetamide. 10 μl of 0.5 mg/ml trypsin was added to each alkylated sample and incubated at 37 °C for 16 h. After desalination, the eluted samples were dried, resuspended with 10 µl 70% formic acid and 340 µl 0.1% trifluoroacetic acid, and the protein concentrations (A260/280) were determined by Nanodrop measurements (Thermo Scientific Nanodrop 2000 UV-Vis Spectrophotometer) for mass spectrometric analysis.

Nano LC-MS/MS analysis was performed on a Thermo Scientific Q Exactive Plus mass spectrometer equipped with a Waters nanoAcquity UPLC system utilizing a binary solvent system. Trapping was performed using a Waters Symmetry^®^ C18 (180 μm × 20 mm) column and peptides were separated using an ACQUITY UPLC PST (BEH) C18 nanoACQUITY Column. MS was acquired in profile mode over the 300-1,500 m/z range and data dependent MS/MS was acquired in centroid mode. Up to 20 MS/MS were collected per MS scan on species with an intensity threshold of 10^4^, charge states 2–6, peptide match preferred, and dynamic exclusion set to 20 seconds.

### Protein identification and compilation of search results

Databases of Sequest (Thermo Fisher Scientific, San Jose, CA, USA; version 2.1.0.81) and X!Tandem (The GPM, thegpm.org; version CYCLONE, 2010.12.01.1) were used for MS/MS samples’ protein identification. Sequest was set up to search against a proteome database including *R. flavipes*, *P. aeruginosa*, and MRSA from NCBI assuming trypsin digestion (https://www.ncbi.nlm.nih.gov/guide/proteins/). X!Tandem was set up to search a subset of the database, also assuming trypsin digestion. Scaffold software (v4.6.1, Proteome Software Inc., Portland, OR) was used to validate MS/MS based peptide and protein identifications. Peptide probabilities from X!Tandem were assigned by the Peptide Prophet algorithm^55^ with Scaffold delta-mass correction. Peptide Probabilities from X!Tandem and Sequest were assigned by the Scaffold Local FDR algorithm. Protein probabilities were assigned by the Protein Prophet algorithm^55^. Protein probabilities and identifications were accepted if they could be established at greater than 95% peptide probability and contained at least two identified peptides to achieve a false discovery rate (FDR; the ratio between the false peptide-spectrum matches (PSM) and the total number of PSMs above the score threshold) less than 5.0%. Proteins sharing significant peptide evidence were grouped into clusters.

### Protein function prediction

We used Blast2GO PRO to search insect protein collections at NCBI and to predict functions of those accepted protein identifications because of the advanced functional analysis to the genomics research of non-model species^56,57^. BLASTp-fast searches were done with an expectation value maximum of 10^−3^.

### Statistical analysis

Generalized linear models (GLM) was used to identify quantities of the accepted proteins across different treatments based on spectral counts. The original protein dataset was first reduced to eliminate proteins that were only present in one replicate with too few spectral counts. The acquired subsets of the protein database consisted of 168 proteins in MRSA-challenged and naïve termites and 177 proteins in *P. aeruginosa*-challenged and naïve termites.

In statistics, GLM is a popular parametric model to fit a given set of empirical observations, i.e., counts data. For our spectral count data, the GLM is expressed as:

$$\mathrm{Log}(Y)={\beta }_{0}+{\beta }_{1}X+\varepsilon ,$$

where *Y* denotes spectral count, *X* stands for treatment and *ε* is the measurement error.

Since our dataset has been verified as overdispersion (i.e., observed variance is higher than the variance of a theoretical model) which is a common characteristic of LC-MS/MS dataset, the quasi-Poisson likelihood method^58^ was used to deal with the overdispersion problem and to increase accuracy. In the following, we give the definition of quasi-Poisson likelihood. For the *i*th response *Y*_*i*_, let E(*Y*_*i*_) = *ui*_,_ Var(*Y*_*i*_) = *φV*(*u*_*i*_), and *U*_*i*_ as ${U}_{i}=\frac{{Y}_{i}-{u}_{i}}{\phi V({u}_{i})}$, *φ* > 0 is the unknown overdispersion parameter. Then a log quasi-likelihood (*Q*_*i*_) for *Y*_*i*_ is defined by the integration of *Ui*: ${Q}_{i}={\int }_{{y}_{i}}^{{u}_{i}}\frac{{y}_{i}-t}{\phi V(t)}dt(t)$, and the log quasi-likelihood for all *n* observations is: $Q={\sum }_{i=1}^{n}{Q}_{i}$, where n = 6 for each comparison. The unknown parameters *β*_0_, *β*_1_, and *φ* can be estimated by many standard statistics software.

As there are over a hundred hypotheses testing between MDR-challenged and naïve termites simultaneously occurred during quasi-Poisson modeling, it is highly likely to get at least one false significant result when running hundreds of hypotheses testing simultaneously. Therefore, finding statistical methods of controlling the rate of false discovery is required. We applied a False Discovery Rate (FDR) method^59^ which was proven to be able to control the FDR by correcting *p*-values when computing from quasi-Poisson likelihood. Specifically, given the problem of testing m null hypotheses simultaneously, denote *H*_*(i)*_ (*i* = 1, 2, …, *m*) the null hypothesis, and *P*_*(i)*_ the corresponding ordered *p*-value satisfying *P*_*(1)*_ ≤ *P*_*(2)*_ ≤ … ≤ *P*_*(m)*_. We rejected all *H*_*(i)*_, *i* = 1, 2, …, *k* and *k* = max{*i*: ${P}_{i}\le \frac{i}{m}\alpha$}, where α is the pre-specified significance level.

Significantly expressed proteins were determined by a FDR corrected quasi *p*-value less than 0.05 and at least 2.5-fold difference in spectral counts. To evaluate variations of hemolymph proteins between *P. aeruginosa-*challenged, MRSA-challenged and naïve termites, and to visualize strong patterns in our dataset, unsupervised hierarchical clustering was performed using Ward’s method with Pearson correlation as similarity metric. This clustering technique organized all data elements into a dendrogram representing the discovered classes. In addition, a principal component analysis (PCA) was applied to the protein expression data to better visualize the dataset after class prediction analysis and the top components were used to illustrate the similarity in protein expression profiles among samples. Hierarchical clustering analysis and PCA was performed on proteins identified as significantly expressed.

All statistical analyses were performed at a significant level of α = 0.05, using statistical software R (https://www.r-project.org/).

### RT-qPCR verification

Total termite RNA from each treatment was obtained with TRIzol Reagent (Thermo Fisher Scientific, San Jose, CA, USA) according to the manufacturer’s instructions and the mRNA was purified with the Oligotex mRNA mini Kit (Qiagen, Valencia, CA, USA). 1 μg of mRNA per sample was reverse transcribed using TaqMan® reverse transcription reagent (Thermo Fisher Scientific, San Jose, CA, USA) to obtain cDNA products and were amplified and the resulting PCR products containing the genes of interest were sequenced by the Laragen Sequencing & Genotyping (Culver City, CA). Using the acquired sequences, specific primers for qPCR were designed using DNASTAR (Madison, WI; Supplementary Table 2). The qPCR reactions were performed with iTaq^TM^ Universal SYBR® Green Supermix on Bio-Rad CFX96 Touch^TM^ Real-Time PCR Detection System (Bio-Rad, Hercules, CA, USA). For each sample, three replicates were performed. The qPCR reactions for *phenoloxidase* and *ferritin* were performed at following condition: 95 °C for 3 min, followed by 40 cycles of 95 °C for 10 s and 54 °C for 30 s. qPCR for the other genes were performed at following condition: 95 °C for 3 min, followed by 40 cycles of 95 °C for 10 s and 58 °C for 30 s. The expressions of 7 genes of MDR-challenged termites were normalized to those of naïve termites and *alpha-tubulin* was used as an internal control. The relative gene expressions were calculated by the method of 2^−ΔΔ^ Ct^2^. The gene expression levels between MDR-challenged termite treatments and naïve termites were analyzed using paired t-tests (n = 9, *α* = 0.05).

## Electronic supplementary material

Supplementary Information
