# Supplementary material for: Hemolymph protein profiles of subterranean termite Reticulitermes flavipes challenged with methicillin resistant Staphylococcus aureus or Pseudomonas aeruginosa
Source: Sci Rep. 2018 Sep 5;8:13251. doi: 10.1038/s41598-018-31681-2 (PMC6125296; doi:10.1038/s41598-018-31681-2)
Supplement: Supplementary file 1 — Supplementary Information [file 41598_2018_31681_MOESM1_ESM.pdf]

Hemolymph protein profiles of subterranean termite *Reticulitermes flavipes* challenged with methicillin resistant *Staphylococcus aureus* or *Pseudomonas aeruginosa*

Yuan Zeng[1#], Xing Ping Hu[1], Guanqun Cao[2], Sang-Jin Suh[3*]

[1]Department of Entomology and Plant Pathology, Auburn University, Auburn, AL, United States of America

[2]Department of Mathematics and Statistics, Auburn University, Auburn University, Auburn, AL, United States of America

[3]Department of Biological Sciences, Auburn University, Auburn University, Auburn, AL, United States of America

*Corresponding author: Sang-Jin Suh, E-mail address: suhsang@auburn.edu (S.-J.S.)

#Current Address: Department of Bioagricultural Sciences and Pest Management, Colorado State University, Fort Collins, CO, Unites States of America

Supplementary Table 1. Reference sequences and primers used for PCR amplification

Protein	Accession Number	Primer sequences (5′→3′)
alpha-tubulin 2	AGM32992.1	Forward: CGCCCGACGTACACGAACTTGA
		Reverse: GCGCCCGGCTCCACAGACT
phenoloxidase	AHB39936.1	Forward: CGCGAGTTACTGACCGAGGAGA
		Reverse: TTTGGGCTGTTAGTGCTGTTGTCA
ferritin	AGM32322.1	Forward: TATGCGCCTTAGGACGGATGAG
		Reverse: TTTGAGGGGTGTTGGGTTCTGA
actin	AGM32156.1	Forward: AGCACCACGTTCAGTCTTTC
		Reverse: GTCACGCACAATTTCTTTCTC
calponin-likey domain containing protein	AGM32561.1	Forward: TAGCCCTGGCAGCATTGGTAAAA
		Reverse: GTGGCGCCCTTGTTCTGTCC
beta-glucosidase	BAO85044.1	Forward: TGCCACAGCCTCTACAAAATCTCG
		Reverse: TCGGCCGCTTCCTCTTCTTCT
		Reverse: CAGGCGGGTTCTTGCACTTATTT
gram-negative binding protein	AEK64796.1	Forward: GGCTTTACGGCGGGTCTCCT
		Reverse: ACGGCGCCATCTTTGTTCC
lysosomal aspartic protease	KDR23365.1	Forward: CAAAGGCCAGTCCAGGTTCA
		Reverse: AGCACGCAGAGCACTACAGGA

Supplementary Table 2. Primers used for RT-qPCR verification

Protein	Accession Number	Primer sequences (5′→3′)
alpha-tubulin 2	AGM32992.1	Forward: AGCCGGCGAACATGATGGTGAAGT
		Reverse: CGTCGGCGGCTGGTAGTTGAT
phenoloxidase	AHB39936.1	Forward: TGGCTACAACGGAATCACT
		Reverse: TCAATACCTCGGGACAGA
ferritin	AGM32322.1	Forward: TGTAGTATTTGCCAGTTGTTA
		Reverse: CTTGCACGGCTTTCATTGTTG
actin	AGM32156.1	Forward: CAAAAGCCAACCGAGAAAA
		Reverse: ACCTGCATCAAAAACAATACCT
beta-glucosidase	BAO85044.1	Forward: TTTGGAGACAGGGTGAAGTGGTG
		Reverse: ATGCCCTGGCGTGTGATAGC
calponin-likey domain containing protein	AGM32561.1	Forward: GAGTGCAAGCGCGATTTTAGTGAG
		Reverse: GTGGCGCCCTTGTTCTGTCC
gram-negative binding protein	AEK64796.1	Forward: CCTGGACGGGGTTTCAATCAA
		Reverse: AACTTCCCAAAATCCACCGTCTG
lysosomal aspartic protease	KDR23365.1	Forward: AGAAAGACTGCCTGAGCCATAC
		Reverse: ACCCCAGAAGTTCCGAGTTGTGT
